# And War Came

**Published:** 1898-05

**Authors:** 


### AND WAR CAME.

WHEN, last month, we wrote of the possibilities of war, still in the hope and belief that it would be averted, we little thought that in spite of the exercise of a wise and conservative diplomacy by the Executive at Washington, hostile batteries would be blazing along the northern coast of Cuba before this edition of the JOURNAL went to press. Nevertheless, such is the fact, and the people of the Republic are thoroughly aroused to all the conditions that belong to the environment of dreadful war.

The time has passed for discussion or argument with reference to the justice of our cause. That has been settled by those whose duty it is to decide such questions. Most of us will quite agree with Mr. Henry Watterson in his brilliant speech at the celebration of General Grant's birthday, in New York, on the 27th of April, when, in an outburst of haughty eloquence, he asserted that

> We want no other warrant for our act of war than the cruel, the heartless story of the Spaniard in America, from the coming of Cortez and Pizarro to the going of Weyler—three centuries of brutality, irradiated only by the pirate's lust for plunder and the tiger's thirst for blood—each succeeding captain-general has seemed to emulate Alva as a rival of Satan by seeking a second immortality of damnation. Before such an array, historical and contemporary, the true American neither consults his geography nor counts the cost. His pulse-beats are the same in Massachusetts and in Mississippi, and whether the band plays "Yankee Doodle" or "Dixie" is all one to him.

Our purpose, however, in introducing the subject at this time is to briefly discuss some of the medical aspects of the war. It seemed at first probable that it would result chiefly in conflicts upon the high seas ; but more recent developments indicate that it will principally be a land war and that the army, rather than the navy, will sustain the chief burden of the conflict. It would appear like good strategy to land a force in Cuba, sufficiently strong to maintain a foothold and to furnish the insurgents with commissary and ordnance supplies, as well as to feed the reconcentrados. If this should prove the policy it will compel Spain to come across the ocean to engage our fleet, and this will put her at a disadvantage with reference to coal and ammunition—two principal commodities in naval warfare.

Our army in Cuba in such event will furnish the chief work for the medical staff, whether from sickness or casualties of battle. Sickness is always the main drawback to the progress or success of an army. It is important that its medical staff shall be composed of efficient men, strong in character, large in experience, skilled in diagnosis and treatment, and equipped with adequate sanitary and hygienic knowledge. It is true they are called surgeons, but it is only exceptionally they are called upon to use the knife, the exercise of which in battle is permitted to only the few of known aptitude for surgical work, and whose surgical judgment can be relied upon. The medical staff of the army will be called upon to exercise the larger functions of physicians every day, but only now and then those of surgeons. How important, then, that the selections for these responsible places should be made with due caution and after careful examinations! In the State of New York, we have no doubt, the state license will be accepted as an evidence of professional proficiency; but we are sure that it becomes the duty of the state to establish a proper medical examination for all other applicants for commissions as medical officers. That this should be done under the direction of the regents of the University is an asseveration that does not permit of justifiable dispute.

Finally, we do not share in the somewhat generally expressed fear of the danger of yellow fever. Surgeon-general Sternberg is an expert regarding the infection of this disease, and Surgeon-general Van Reypen has also had large experience in its management. Supervising Surgeon-general Wyman is also at the head of a medical corps containing many experts on the infection and management of yellow fever. The combined skill of these men will be brought to bear on the proper sanitation and hygienic care of our military and naval forces in and about Cuba. The proper selection of camp sites, good water supply, ample drainage, personal hygiene, the isolation of infected cases—all these will deserve and receive the most skilful and serious attention of the constituted heads of the medical departments of the three services. Let the government at Washington see that only competent subordinates are commissioned to support them and to execute their orders, and then we believe there will be no serious inroads made upon our forces by yellow fever or any other calamitous disease.

THE BUFFALO EXPRESS very properly advocates the abatement of street nuisances, such as peddlers, organ grinders, beggars and canvassers. We quite agree with our contemporary, and would suggest as an amendment to its plaint, that a city, no matter how fortunately situated as regards geography, cannot make solid progress unless it keeps its streets clean and suppresses unnecessary noises. Both of these conditions are so essential to health and comfort, that desirable permanent residents will not be attracted to a city that does not scrupulously maintain them. It is far from pleasant to be aroused at half-past four or five o'clock in the morning twice a week, by the rattling trucks of the garbage collectors that invade one's premises when sleep is sweetest. Furthermore, it is inexcusable for a city to add to the interminable din of the early morning factory whistles by permitting its fire tug to belch forth its unearthly screeches, the only purpose of which can be to delight the appetite of its officers for noise.

